# The Popular Science Monthly

**Published:** 1878-01

**Authors:** 


The January number of THE POPULAR SCIENCE MONTHLY opens with the third of Prof. R. H. Thurston's series of papers on "The Growth of the Steam-Engine." This, like the two previous articles, is profusely illustrated with very fine woodcuts, and is devoted to a history of the Development of the Locomotive. The second article is on "Health-Matters in Japan," by Prof. Edward S. Morse, who has just returned from a visit to that country. He describes a system of practical hygiene in operation in the cities of Japan that should make Western nations ashamed of their boasted civilization. "How to make our Ideas clear" is the subject of Prof. C. S. Peirce's second paper on "Illustrations of the Logic of Science." "The Archer-Fishes" is an illustrated article concerning a group of curious marine forms that capture their insect prey by violently squirting or shooting at it drops of water from their long, slender mouths or beaks. "Temperaments," by Dr. Ely Van De Warker, is a very interesting and, at the same time, scientific article on a subject that has been much written about, but little understood.

This magazine, now nearly six years old, started as an experiment, quickly took a bold but dignified position; and, without resorting to holiday spurts or sensationalism of any kind, has attained a remarkable success, both in circulation and in the influence it exerts on thinking people. Those who are unable or unwilling to think had better let it alone, as it has a merciless disrespect for the hide-bound torpidity it is doing so much to remove. New York: D. Appleton & Co. Five dollars per year, 50 cents per copy.

———:o:———

DR. BULKLEY is giving a course of free lectures on Diseases of the Skin, at Demilt Dispensary, New York City, Saturday afternoons from 2 to 3 o'clock. The course began Dec. 1st, and will prove of great interest to the student.

